# How chloroplasts protect themselves from unfolded proteins

**DOI:** 10.7554/eLife.51430

**Published:** 2019-10-15

**Authors:** Felix Kessler, Paolo Longoni

**Affiliations:** Laboratory of Plant PhysiologyUniversity of NeuchâtelNeuchâtelSwitzerland

**Keywords:** protein homeostasis, chloroplast, photoprotection, organelles, cell signaling, *Chlamydomonas reinhardtii*

## Abstract

A genetic screen has identified the first signaling component of the unfolded protein response in chloroplasts.

**Related research article** Perlaza K, Toutkoushian H, Boone M, Lam M, Iwai M, Jonikas MC, Walter P, Ramundo S. 2019. The Mars1 kinase confers photoprotection through signaling in the chloroplast unfolded protein response. *eLife* **8**:e49577. doi: 10.7554/eLife.49577

Eukaryotic cells contain organelles that perform specific roles. The chloroplasts in algae and plants, for example, harvest the energy from sunlight to produce organic molecules via photosynthesis. An organelle must import proteins from the cytoplasm to perform its role: however, these proteins have to be unfolded when they are imported, so they must be rapidly processed and folded once they are inside the organelle. If proteins that are not correctly folded accumulate, they can become toxic, and must be detected and removed in order to protect the organelle from damage.

A quality control system consisting of small heat shock proteins, chaperones and proteases ensures that all proteins entering the chloroplast are properly folded. However, when algae are exposed to high levels of light, or when a protease called ClpP1 is missing, this quality control system can become compromised and overwhelmed by unfolded proteins. This causes the chloroplast to trigger an ‘unfolded protein response’ (UPR) that upregulates the production of various proteins in the nucleus, which then travel to the chloroplast to help it cope with this overload of unfolded proteins. Although the UPR is well-understood in other organelles, such as the ER and mitochondria, the UPR in chloroplasts has been described to a far lesser degree. For example, it remains unclear how the chloroplast monitors the level of unfolded proteins and communicates this information to the nucleus.

Now, in eLife, Silvia Ramundo of the University of California San Francisco (UCSF) and colleagues – including Karina Perlaza as first author – report how they used a genetic screen to discover the first signaling component of chloroplast unfolded protein response (cpUPR) in the algae *Chlamydomonas reinhardti* (Perlaza et al., 2019). To create the screen, the researchers used an inducible switch that blocks the production of the ClpP1 protease: this blocking causes unfolded proteins to accumulate inside the chloroplast, triggering the cpUPR (Figure 1; Ramundo et al., 2014; Ramundo and Rochaix, 2014). To monitor the response, algae were engineered to express a fluorescent reporter that was under the control of a nuclear expressed gene that only becomes upregulated when the cpUPR is switched on. Perlaza et al. then inserted random mutations into the genome of these engineered algae, and screened for mutants that were no longer displaying fluorescence despite having activated the cpUPR (Figure 1).

**Figure 1.**
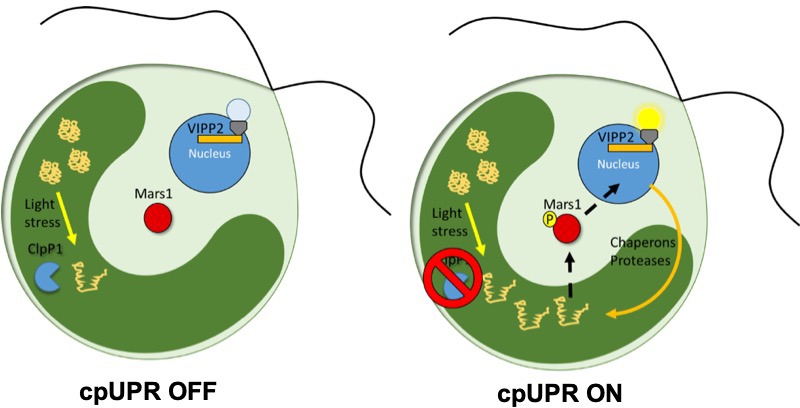
The unfolded protein response in chloroplasts. In wild-type *C. reinhardti* algae a protease called ClpP1 is responsible for removing unfolded proteins produced by stress (left). The loss of ClpP1 (right) leads to the accumulation of unfolded proteins in the chloroplast (dark green) triggering the cpUPR. This causes nuclear genes which encode factors that assist in protein folding (such as chaperones and proteases) to become upregulated. In order to monitor changes to cpUPR signaling, a fluorescent protein was put under the control of one of these factors (VIPP2), so that fluorescence could only be observed when cpUPR was induced. By inserting random mutations into the genome and screening for mutants no longer displaying the fluorescent reporter, Perlaza et al. were able to identify MARS1, a kinase involved in transmitting the cpUPR signal from the chloroplast to the nucleus. MARS1: mutant affected in chloroplast-to-nucleus retrograde signaling 1.

Using this screen, the team – which includes researchers from UCSF, the Lawrence Berkeley Lab and Princeton – identified a gene coding for a protein called MARS1 that is essential for activating the cpUPR. Because MARS1 was localized outside of the chloroplast and contained a kinase domain (a critical component of cell signaling), Perlaza et al. deduced that MARS1 is likely to be involved in sending the cpUPR signal from the chloroplast to the nucleus. Furthermore, mutating the catalytic residue within the kinase domain revealed that kinase activity was critical for the cpUPR.

Stress caused by high levels of sunlight leads to an increased production of reactive oxygen species (ROS) that damage proteins inside the chloroplast, which in turn triggers the cpUPR. Perlaza et al. found that mutating the kinase domain of MARS1 caused algae to be more susceptible to this type of chloroplast stress. Notably, the increased susceptibility of the mutants was only linked to chloroplast stressors that trigger the cpUPR, suggesting that MARS1 is specific for the unfolded protein response. Perlaza et al. also observed that mutant algae that had a dominantly active version of the kinase displayed enhanced resistance to high levels of light and ROS. This opens a potential avenue for how the cpUPR could be exploited to increase the efficiency of photosynthesis in other organisms.

The study of cpUPR is still in its infancy, but it is already revealing its importance in chloroplast development and the protection of photosynthetic activity. A crucial next step will be to apply this knowledge to higher plants, where MARS1 has not been identified but where responses equivalent to cpUPR have been shown to exist (Llamas et al., 2017). Any new components of the cpUPR pathway that researchers are able to identify could be potential new targets for increasing yields in crop species.
